# Relative Importance of Stochastic Assembly Process of Membrane Biofilm Increased as Biofilm Aged

**DOI:** 10.3389/fmicb.2021.708531

**Published:** 2021-09-10

**Authors:** Gerald K. Matar, Muhammad Ali, Samik Bagchi, Suzana Nunes, Wen-Tso Liu, Pascal E. Saikaly

**Affiliations:** ^1^Biological and Environmental Science and Engineering Division, Water Desalination and Reuse Research Center, King Abdullah University of Science and Technology, Thuwal, Saudi Arabia; ^2^Biological and Environmental Science and Engineering Division, Advanced Membranes and Porous Materials Center, King Abdullah University of Science and Technology, Thuwal, Saudi Arabia; ^3^3207 Newmark Civil Engineering Laboratory, Department of Civil and Environmental Engineering, University of Illinois at Urbana-Champaign, Urbana, IL, United States

**Keywords:** membrane bioreactor, biofouling, hydrophobic membranes, hydrophilic membranes, biofilm community assembly, ecological processes

## Abstract

The relative importance of different ecological processes controlling biofilm community assembly over time on membranes with different surface characteristics has never been investigated in membrane bioreactors (MBRs). In this study, five ultrafiltration hollow-fiber membranes – having identical nominal pore size (0.1μm) but different hydrophobic or hydrophilic surface characteristics – were operated simultaneously in the same MBR tank with a constant flux of 10 liters per square meter per hour (LMH). In parallel, membrane modules operated without permeate flux (0 LMH) were submerged in the same MBR tank, to investigate the passive microbial adsorption onto different hydrophobic or hydrophilic membranes. Samples from the membrane biofilm were collected after 1, 10, 20, and 30days of continuous filtration. The membrane biofilm microbiome were investigated using 16S rRNA gene amplicon sequencing from DNA and cDNA samples. Similar beta diversity trends were observed for both DNA- and cDNA-based analyses. Beta diversity analyses revealed that the nature of the membrane surface (i.e., hydrophobic *vs*. hydrophilic) did not seem to have an effect in shaping the bacterial community, and a similar biofilm microbiome evolved for all types of membranes. Similarly, membrane modules operated with and without permeate flux did not significantly influence alpha and beta diversity of the membrane biofilm. Nevertheless, different-aged membrane biofilm samples exhibited significant differences. Proteobacteria was the most dominant phylum in early-stage membrane biofilm after 1 and 10days of filtration. Subsequently, the relative reads abundance of the phyla Bacteroidetes and Firmicutes increased within the membrane biofilm communities after 20 and 30days of filtration, possibly due to successional steps that lead to the formation of a relatively aged biofilm. Our findings indicate distinct membrane biofilm assembly patterns with different-aged biofilm. Ecological null model analyses revealed that the assembly of early-stage biofilm community developed after 1 and 10days of filtration was mainly governed by homogenous selection. As the biofilm aged (days 20 and 30), stochastic processes (e.g., ecological drift) started to become important in shaping the assembly of biofilm community.

## Introduction

Biofouling is still considered to be the “Achilles Heel” of membrane-based processes (Flemming et al., 2011), particularly in membrane bioreactors (MBRs; Hai et al., 2019). The accumulation of membrane foulants intensifies the frequency of physical and chemical cleanings (Malaeb et al., 2013; Vanysacker et al., 2014), and the replacement of membrane modules becomes inevitable in the event of irreversible fouling (Le-clech et al., 2006). Several control strategies have been proposed to reduce biofouling in MBRs, including physical and chemical cleaning, membrane surface modification (e.g., charge, hydrophobicity, roughness; Lee et al., 2014; Zhang et al., 2015), and biological-based antifouling strategies (e.g., quorum quenching, enzymatic disruption, energy uncoupling; Malaeb et al., 2013).

Membrane surface modification acquired considerable attention to control or limit membrane biofouling. For instance, polytetrafluoroethylene membranes grafted with carbon nanotube were operated as electric conductive membranes, which reduced the biomass accumulation on the surfaces of the modified membranes (Jiang et al., 2019). Reverse osmosis (RO) membranes modified with polydopamine exhibited an enhanced antifouling capabilities during 25h of continuous filtration (Baek et al., 2017). Quorum sensing inhibitors (QSI) molecules were embedded onto the surfaces of commercial chlorinated polyethylene membranes (Tse et al., 2018) and polyamide RO membranes (Kim et al., 2021), and both studies confirmed the effectiveness of grafting QSI molecules on membrane surfaces to reduce biofouling. Surface modifications with zwitterionic functionalization have been successfully demonstrated to enhance the fouling resistance of RO and forward osmosis membranes (Tirado et al., 2016; Duong et al., 2019). Finally, the introduction of silver as a biocide has been long explored as an anti-fouling strategy (Madhavan et al., 2014; Nunes, 2020; Pejman et al., 2020). Despite these numerous studies that confirmed the positive impact of surface modification in avoiding membrane biofouling, the investigation on how physicochemical properties of different membrane surfaces influence the membrane biofilm community assembly pattern is still lacking. In contrast to abiotic fouling (i.e., scaling, organic, and particle fouling), biofouling is caused by microorganisms that can attach, multiply, and grow into a mature biofilm (Flemming et al., 2011).

Membrane biofilm formation involves several successional steps, including an initial conditioning layer that covers the membrane surface, modifies its surface characteristics, and renders it more favorable for the attachment of early colonizers, and the development of relatively aged biofilm (Matar et al., 2017). The relative importance of different community assembly mechanisms during the sequential steps of biofilm formation on membranes with different surface characteristics has not been studied. Moreover, only a few studies examined biofilm microbial community assembly processes on identical membrane surfaces (Matar et al., 2017; Xu et al., 2019). In one study it has been reported that the community assembly of membrane biofilm (early and aged) in full-scale MBRs with identical membrane type was not stochastic (Matar et al., 2017), and specific local conditions may have been responsible for the selection of biofilm communities from the suspended biomass. In another study, it was shown that identical membranes tested with a low-flux of 8 liters per square meter per hour (LMH) harbored a membrane biofilm with much lower diversity than identical membranes operated with a higher flux of 16 LMH (Xu et al., 2019). It was further revealed that the relative importance of the ecological stochastic process was higher in the high-flux membrane biofilm community because of the higher stochastic deposition of bacterial cells from suspended biomass due to the stronger convective force (Xu et al., 2019). Nevertheless, the relative importance of various biofilm community assembly processes over time and on membranes with different surface characteristics was not quantified before. These processes for community assembly include both stochastic and deterministic mechanisms. Stochastic processes include ecological drift driven by random birth-death events and random colonization (Zhou and Ning, 2017). Conversely, deterministic processes are caused by both abiotic (environmental filtering: changes in pH, temperature, salinity, etc.) and biotic factors (competition, facilitation, mutualism, predation, etc.; Ali et al., 2019; Ning et al., 2019). Moreover, several distinct mechanisms can simultaneously act on the microbiome, especially in complex communities – and that the relative proportion of these processes can change (Stegen et al., 2012, 2013; Vass et al., 2020). Using a combination of several approaches and ecological frameworks, it is now possible to resolve the dynamics of assembly processes over space and time (Stegen et al., 2013, 2015; Ning et al., 2020). The use of null models has continued to gain attention to identify and quantify community assembly in natural (Stegen et al., 2012, 2013; Vass et al., 2020) and engineered ecosystems (Matar et al., 2017; Ali et al., 2019; Xu et al., 2019).

The objectives of the current study were to characterize the microbial community structure of biofilm on the surfaces of five different polymeric ultrafiltration hollow-fiber membranes (HFMs) having different hydrophobic and hydrophilic surface properties, and to assess the relative importance of different biofilm community assembly processes over time on the five different types of membranes. The null model was applied to quantify the relative importance of deterministic and stochastic processes in the biofilm microbial assembly on different membrane surfaces. Five hydrophobic or hydrophilic membranes that differed in surface characteristics were operated simultaneously in the same MBR tank using a low flux of 10 LMH. Low flux values have been reported to reduce and/or delay membrane fouling due to the low convection of foulants (including bacterial cells; Judd, 2008). In parallel, identical membrane modules were inserted in the same MBR tank and operated under static mode (without filtration) to investigate membrane biofilm community assembly without the effect of convection. It is also pertinent to mention that the previous studies on membrane biofilm community assembly were mainly based on 16S rRNA gene amplicon sequencing. In this study, microbiome composition and assembly were analyzed using 16S rRNA and 16S rRNA gene amplicon sequencing.

## Results

### Performance of the Lab-Scale MBR

The MBR was fed with synthetic wastewater with a concentration of 404±8.6mg/l chemical oxygen demand (COD) and 46±1.5mg/l ${\mathrm{NH}}_{4}^{+}‐N$ . The concentrations of COD, ${\mathrm{NH}}_{4}^{+}$ , ${\mathrm{NO}}_{2}^{-}$ , and ${\mathrm{NO}}_{3}^{-}$ were measured during the acclimatization and experimental phases (Supplementary Figure S1). The performance of the MBR during the experimental phase was stable with average removal of COD, ${\mathrm{NH}}_{4}^{+}‐N$ , and total nitrogen of 91.9±2.8%, 86.58±2.25%, and 85.9±6.2%, respectively. The effluent concentration of ${\mathrm{NO}}_{2}^{-}$ and ${\mathrm{NO}}_{3}^{-}$ during the experimental phase was 0.17±0.09 and 0.12±0.13mg/l, respectively.

The trans-membrane pressure (TMP) was continuously monitored throughout the experimental phase for the five different membrane types (Supplementary Figure S2). After 1day of filtration, the hydrophobic membranes (polyoxadiazole, POX and polytriazole, PTA) exhibited a higher TMP (80kPa) compared to the hydrophilic membranes (sulfonated polytriazole, SPTA 44kPa, and sulfonared polysulfone, SPSU 20kPa) due to the high hydrophobic surface character, which favors the adhesion of hydrophobic solutes leading to a lower effective permeation and higher TMP values (Supplementary Figure S2). The TMPs remained at this level until the end of the experiment. The commercial membrane exhibited the lowest TMP that reached 20kPa after 12days of operation and remained at this level until the end of the experiment. No chemical or physical cleaning was performed during the experimental phase.

### Microbial Community Structure and Composition

A total of 88 16S rRNA gene samples from the reactor were sequenced, including 44 samples each for DNA and cDNA. These samples were taken at 1, 10, 20, and 30days of reactor operation, and included each type of membrane [POX, PTA, SPTA, SPSU, and commercial poly(vinylidene fluoride; PVDF), COM] and mixed liquor suspended solids (MLSS). The DNA sample of MLSS collected on day 10 was excluded from the analysis as it produced a very low number of reads. The minimum and maximum number of non-chimeric, quality-filtered reads were 1,392 and 18,070, respectively, with 803,028 reads in total (Supplementary Table S1). The non-chimeric, quality-filtered reads were clustered into 480 operational taxonomic units (OTUs) at 97% identity. The samples were rarefied to an even number of reads (4,386) per sample (Supplementary Figure S3). Six samples had less than 4,386 reads and were excluded from the dataset after rarefication to even sequencing depth.

**Figure 1:**
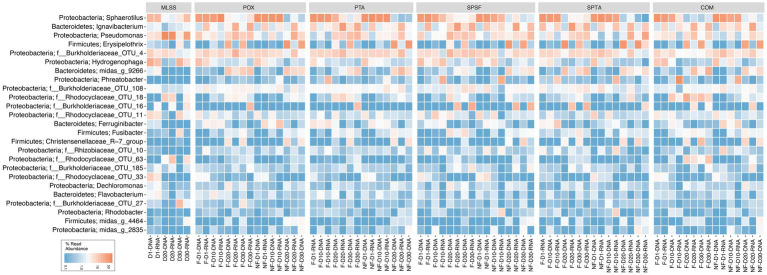
Heatmap distribution of the top 25 taxa classified down to genus-level or the lowest classifiable taxonomic level (f, o, c, and p represent family, order, class, and phylum, respectively) derived from membrane biofilms and mixed liquor samples. The color intensity in each cell reflects relative reads abundance of taxa in the corresponding sample. POX (polyoxadiazole), PTA (polytriazole), SPTA (sulfonated polytriazole), SPSU (sulfonated polysulfone), and COM (commercial PVDF membrane) membranes were operated under 10 LMH flux (F) and without permeate flux (NF). Mixed liquor suspended solids (MLSS) corresponds to the mixed liquor suspended solids sample. D1, D10, D20, and D30 refer to sampling days 1, 10, 20, and 30, respectively. DNA and RNA represent 16S rRNA gene amplicon sequencing dataset from DNA and cDNA samples, respectively. The heatmap generated was based on a rarefied dataset sub-sampled to 4,386 sequences per sample. The dataset used in this analysis includes both flux and no flux conditions.

A heatmap was generated from a rarefied dataset to present the relative reads abundance of the microbial communities classified at the genus-level or lowest classifiable taxonomic level (Figure 1). The phyla Proteobacteria, Firmicutes, and Bacteroidetes were found abundant within the membrane biofilm and the MLSS. The genus *Sphaerotilus*, *Ignavibacterium*, *Pseudomonas*, *Erysipelothrix*, *Hydrogenophaga*, and OTU_4 (belonging to the *Burkholderiaceae* family) were found to be the most abundant in the samples. It is pertinent to mention that bacteria belonging to the genus *Sphaerotilus* and *Hydrogenophaga* were progressively decreased, and other genera such as *Ignavibacterium* and *Pseudomonas* increased over time. Notably, *Erysipelothrix* (belonging to phylum Firmicutes) also increased in relative reads abundance at days 20 and 30 in the biofilm samples.

### Flux, Membrane Material, and Surface Characteristic Do Not Influence Alpha and Beta Diversity

Alpha diversity measurements were performed for the different membrane biofilms and MLSS samples (Supplementary Figure S4). Diversity values across different samples extracted from the five different membranes and MLSS samples ranged as follows: observed OTUs (58–239), Shannon (1.66–3.92), Simpson (0.58–0.95), and Chao 1 (69.4–285.2; Supplementary Figure S4). The observed OTUs and Chao 1 species richness indices were significantly different between MLSS and biofilm samples (value of *p* <0.05). However, alpha diversity was not significantly different between the different membrane materials, and membranes modules operated with and without flux (value of *p* >0.05).

Non-metric multidimensional scaling (NMDS) analysis using taxonomic (Bray-Curtis) distance metric and Principal Coordinates Analysis (PCoA) using phylogenetic (Unweighted UniFrac) distance metric were performed for different membrane materials (Supplementary Figure S5), surface characteristic (Supplementary Figure S6), and between membrane modules operated with (10 LMH) and without (0 LMH) flux (Supplementary Figure S7). These ordination plots (Supplementary Figure S5–S7) suggest that the membrane type, surface characteristics (hydrophobicity or hydrophilicity), and the imposed operating condition (with 10 LMH flux or without flux 0 LMH) did not affect the composition of the membrane biofilm communities. This was observed in both DNA-based and cDNA-based communities.

### Membrane Biofilm Communities Evolve Over Time

**Figure 2:**
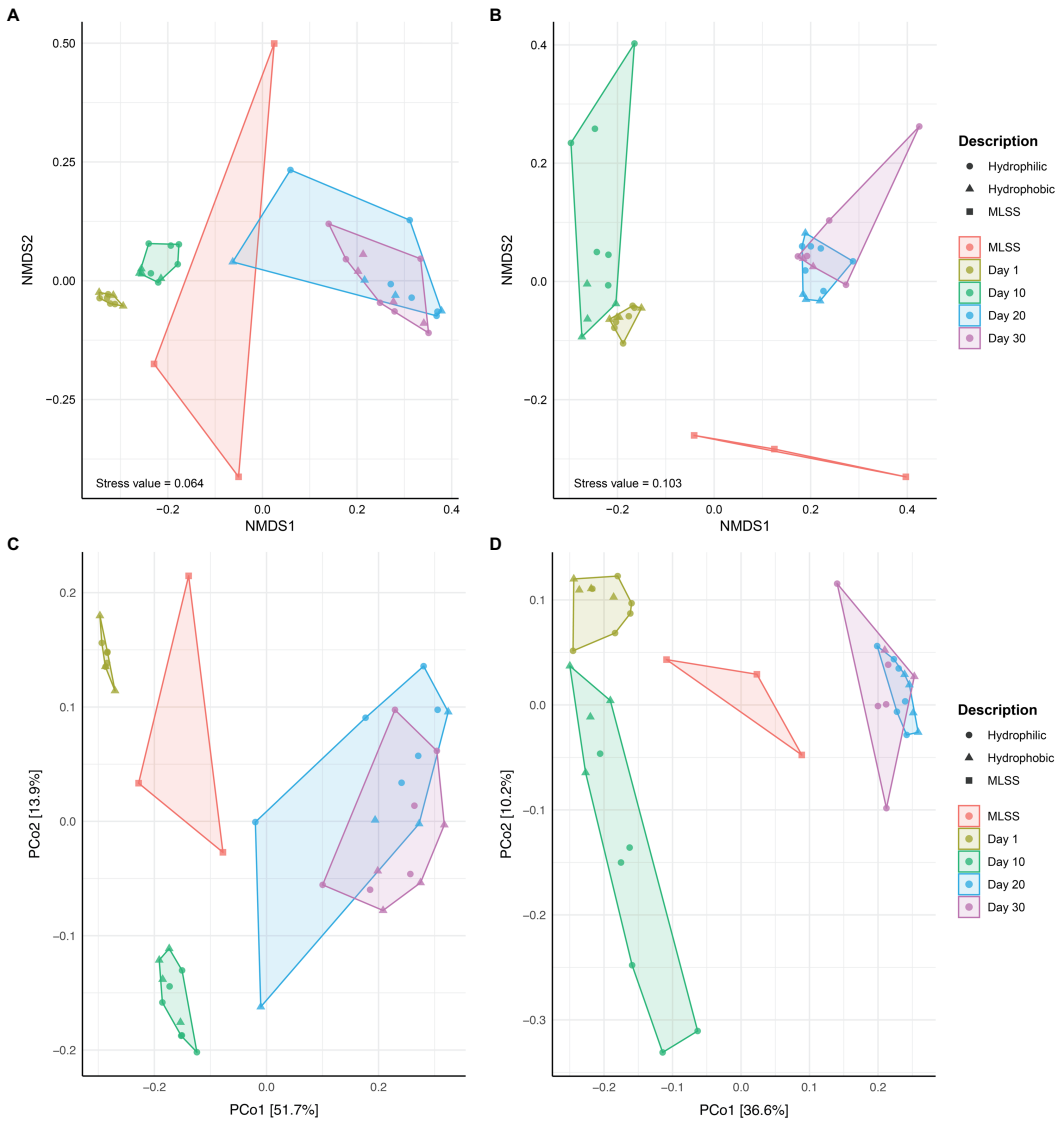
The ordination plots of MLSS and different-aged membrane biofilm samples. Non-metric multidimensional scaling analysis based on Bray-Curtis **(A,B)** and Principal Coordinates Analysis (PCoA) based on Unweighted UniFrac **(C,D)** distance metrics. Microbial community diversity according to variances in 16S rRNA gene dataset from DNA **(A,C)** and cDNA **(B,D)** samples. These plots were generated based on a rarefied dataset sub-sampled to 4,386 sequences per sample. The dataset used in this analysis includes both flux and no flux conditions.

The NMDS and PCoA analysis revealed differences in community composition between MLSS and membrane biofilm samples at different times of sampling (Figure 2). Membrane biofilm samples from days 1 and 10 clustered into two distinct groups, while all membrane biofilm samples from days 20 and 30 clustered into one group (Figures 2A,C). Similar results were found in the ordination plots drawn with the cDNA-based dataset (Figures 2B,D). Permutational multivariate analysis of variance (ADONIS) was used to determine if the dissimilarity in the bacterial community composition between different-aged biofilms was statistically different (Supplementary Table S2). The dissimilarity between the early biofilm community (i.e., day 1) and different-aged membrane biofilm communities increased with the age of the biofilm, and R^2^ ADONIS statistically increased with the increase in biofilm age.

### Enrichment of Specific Phyla and Genera as the Biofilm Ages

**Figure 3:**
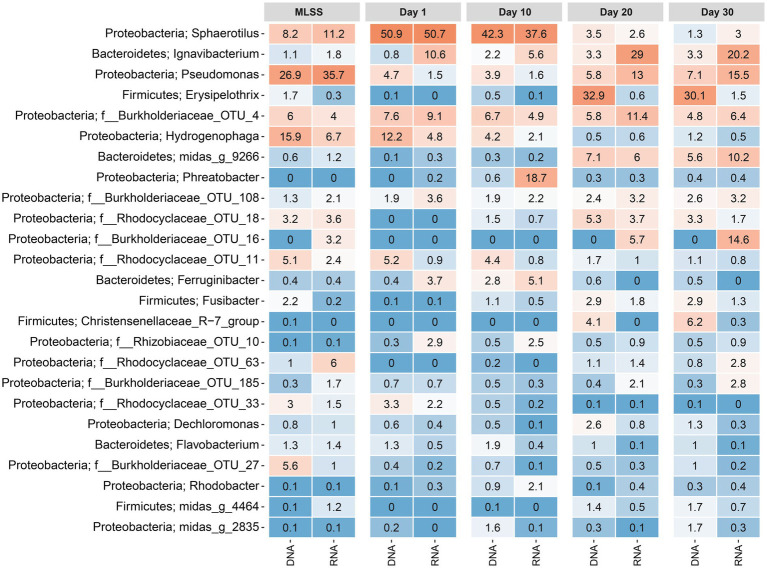
Heatmap distribution of the top 25 taxa classified down to genus-level or the lowest classifiable taxonomic level (f, o, c, and p represent genus, family, order, class, and phylum, respectively) derived from average relative reads abundance for mixed liquor, suspended sludge, and membrane biofilms. MLSS corresponds to the mixed liquor suspended solids sample. Day 1, Day 10, Day 20, and Day 30 refer to sampling days 1, 10, 20, and 30, respectively. The color intensity in each cell reflects relative reads abundance of taxa in the corresponding sample. Similarly, DNA and RNA represent the 16S rRNA amplicon sequencing dataset from DNA and cDNA, respectively. The heatmap was generated based on a rarefied dataset sub-sampled to 4,386 sequences per sample. The ampvis2 package was used to generate the heatmap by averaging the relative abundances of all the samples (i.e., different materials with and without flux) for each sampling time.

A heatmap was plotted, showing the average relative reads abundance and phylogenetic classification of OTUs, in order to visualize microbial communities in the MLSS and temporal dynamics of the taxa in different-aged membrane biofilms (Figure 3). On day 1, the genus *Sphaerotilus* was the most abundant (51% based on DNA and RNA) followed by *Hydrogenophaga* (12% based on DNA and 5% based on RNA) and OTU_4 belonging to the family *Burkholderiaceae* (8% based on DNA and 9% based on RNA; Figure 3). However, the relative reads abundances of *Sphaerotilus* and OTU_4 (*f__Burkholderiaceae*) decreased to 3.5% based on DNA and 2.6% based on RNA and 5.8% based on DNA and 11% based on RNA after 20days of filtration. The relative reads abundance of *Hydrogenophaga* (class Betaproteobacteria) decreased with time to only 1% based on DNA and RNA on day 30. These taxa mentioned above belonged to the phylum Proteobacteria. Interestingly, three genera that belong to the phylum Firmicutes were barely detected in early membrane biofilm at days 1 and 10, yet they dominated the microbial community of relatively aged membrane biofilm at days 20 and 30. For instance, the DNA-based relative read abundance of *Erysipelothrix*, *Fusibacter*, and *Christensenellaceae_R−7_group* increased to 30% (1.5% RNA-based relative read abundance), 3% (1.3% RNA-based), and 6% (0.3% RNA-based), respectively in the membrane biofilms after 30days of filtration. The phylum-level heatmap depicted that the relative read abundance of Proteobacteria progressively decreased from 93% (DNA-based) and 81% (RNA-based) on day 1 to 35% (DNA-based) and 61% (RNA-based) on day 30 (Supplementary Figure S8). On the other hand, the DNA-based relative read abundance of phyla Bacteroidetes and Firmicutes were increased from 4% (17% RNA-based relative read abundance) and 0.2% (0.1% RNA-based; day 1) to 17% (32% RNA-based) and 44% (5% RNA-based; day 30), respectively. A clear and identical temporal dynamic was noted within membrane biofilms developed with time on the five different membranes. These results suggest that the biofouling communities on the five different membranes created a favorable niche that evolved taxa belonging to the phyla Bacteroidetes and Firmicutes.

### A Quantitative Framework Revealed That the Dominant Ecological Process for Membrane Biofilm Community Assembly Varied With the Age of the Biofilm

**Figure 4:**
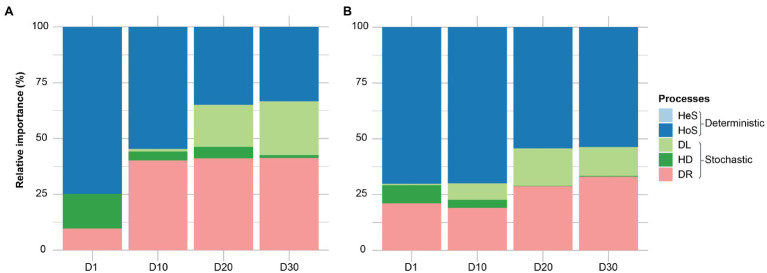
The relative importance of different ecological processes involved in community assembly of membrane biofilms. Panels **(A)** and **(B)** represent the 16S rRNA amplicon sequencing dataset from DNA and cDNA, respectively. Here, “processes” particularly means community assembly processes, including heterogeneous selection (HeS), homogeneous selection (HoS), homogenizing dispersal (HD), dispersal limitation (DL), and ‘drift’ (DR). The definitions of these ecological processes related to community assembly are adopted from (Zhou and Ning, 2017). HeS refers to the selection process under homogeneous abiotic and biotic environmental conditions leading to more similar structures among communities. HoS refers to the selection process under heterogeneous abiotic and biotic environmental conditions leading to more dissimilar structures among communities. HD represents a very high rate of dispersal among communities, which homogenizes the communities such that their structures are very similar. DL is caused by the restriction in movement of individuals to and/or establishment of individuals (colonization) in a new location, leading to more dissimilar structures among communities. DR represents random changes, with respect to species identity, in the relative abundances of different species within a community over time due to the inherent stochastic processes of birth, death, and reproduction. D1, D10, D20, and D30 refer to sampling days 1, 10, 20, and 30, respectively. The dataset used in this analysis includes both flux and no flux conditions.

It is generally accepted that selection, dispersal, diversification, and drift are major community assembly processes. However, it is always challenging to dissect the contribution of each ecological process in community assembly. A quantitative framework (iCAMP; Ning et al., 2020) approach was used to determine the temporal dynamics of each ecological process in community assembly of membrane biofilms. Homogenizing selection – when stable environmental conditions result in consistent selective pressure – was the dominant assembly mechanism in all categories, ranging from 33 to 74% (Figure 4). The ecological drift (stochastic processes: random birth-death events and random dispersal) accounted for 9–41% of community turnover. Heterogeneous selection played no role, which is expected because of the identical environmental conditions of the samples (same temperature, pH, SRT, etc.). Apparent differences were measured in the temporal dynamic of membrane biofilm assembly processes, as the relative importance of homogeneous selection decreased with time and drift increased with time for DNA- and cDNA-based datasets. Dispersal limitation in membrane biofilm increased in relative importance from 0 to 23% with the age of membrane biofilm. Dispersal limitation occurs when reduced colonization (immigration) leads to more dissimilar communities and is likely the underlying ecological mechanism contributing to change in relatively aged (days 20 and 30) biofilm categories as it was noted in the beta diversity ordination plot (Figure 2). A similar trend of ecological assembly mechanisms was observed for both DNA-based and cDNA-based communities.

## Discussion

Membrane biofilms represent a metabolically active and structurally complex component in biofouling and previous studies mainly focused on the diversity and community composition of membrane biofilms. However, little is known about the processes (deterministic and/or stochastic) that control biofilm community assembly over time on membranes with different surface characteristics. Here, we assessed the patterns of bacterial community composition in membrane biofilms developed on different hydrophobic or hydrophilic membranes operated with and without flux, and the assembly processes that determine these patterns over time.

Earlier studies in the marine environment assessing membrane biofilm diversity on hydrophobic and hydrophilic surfaces revealed that the material’s physicochemical properties influenced the attachment of early colonizers and the subsequent microbial community composition (Dang and Lovell, 2000; Grasland et al., 2010). In the current study, a similar microbial community evolved (Figure 2) onto the different HFM surfaces modified into a more hydrophobic or hydrophilic character (Supplementary Figure S6), possibly due to the conditioning layer that could have quickly covered the membrane surfaces in a nutrient-rich environment (such as MLSS) as opposed to oligotrophic environments (seawater). Our results are also in line with our previous study comparing the same four hydrophobic and hydrophilic membranes used in the current study, on which a conditioning extracellular polymeric substances (EPS) layer – rich in proteins – was developed at the early stage of filtration (day 1) and evolved subsequently into an identical EPS composed mainly of polysaccharides during the later stages of MBR filtration (Matar et al., 2016). The biofilm biopolymers clustered according to the sampling event (time) regardless of the membrane surface chemistry (hydrophobic or hydrophilic) or operating mode (with or without permeate flux; Matar et al., 2016). It has been reported that membrane surface roughness is more important than other membrane properties (hydrophobicity/hydrophilicity and surface charge) in shaping the biofilm communities on different membrane types immersed in wastewater and operated without permeate flux production (passive adsorption; 0 LMH; Lee et al., 2014). In the current study, no remarkable distinction was observed in the membrane biofilm communities between membranes operated with flux (10 LMH) or without permeate flux (0 LMH; Supplementary Figure S5). The imposed permeate flux (10 LMH) was low enough to eliminate any possible effect due to the convective force that pulls bacterial cells towards the membrane surface at high permeate flux. Consequently, passive adsorption and bacterial attachment could have occurred equally on all the membranes operated with flux (10 LMH) and without permeate flux (0 LMH). In an earlier study, it was shown that the composition of membrane biofilm communities from two MBRs operating at an SRT of 8 for 30days were very similar at low flux (15 LMH) and when the imposed permeate flux was increased to 30 LMH, the biofilm communities that developed on the membrane surfaces of the MBRs operating at different SRTs were very distinct (Huang et al., 2008). Taken together, these results suggest that membrane surface modifications that cause the membrane to become more hydrophobic or hydrophilic are not a key determinant in shaping the biofilm microbial community structure.

Successional biofilm development including attachment of early colonizers and growth into relatively aged biofilm occurred reproducibly on the five membrane types (Figure 2). Also, the biofilm communities evolved differently compared to the MLSS community (Figures 2, 3). Previous lab- and full-scale MBR studies reported dissimilarity in the community structure between MLSS and membrane biofilm communities (Luo et al., 2017; Matar et al., 2017; Xu et al., 2019). In the current study, *Sphaerotilus* (Phylum: Proteobacteria and Class: Betaproteobacteria) dominated the composition of all membrane biofilms at days 1 and 10, but then significantly decreased in relative abundance at days 20 and 30. Similarly, *Hydrogenophaga* was dominant in the membrane biofilms at day 1, but then gradually decreased in abundance as the biofilm aged (Figure 3). *Hydrogenophaga* is an autotrophic H_2_-oxidizing bacteria and was reported to play an important role in denitrification processes (Hoshino et al., 2005; Yoon et al., 2009). Our MBR was operated under intermittent aeration with 30min aeration followed by 30min anoxic cycles to achieve simultaneous carbon and complete nitrogen removal and enrichment of the sludge (i.e., MLSS) with *Hydrogenophaga* (Figure 3). It is pertinent to mention that the dominant populations in MLSS, e.g., *Sphaerotilus*, *Hydrogenophaga*, *Rhodocyclaceae_OTU_11*, were also the dominant initial colonizers on membrane surfaces. Later, initial colonizers were replaced by metabolically versatile heterotrophs belonging to phylum Firmicutes, which dominated (44.1%) the composition of the membrane biofilms as the biofilm aged (Supplementary Figure S8), despite its low relative abundance in MLSS (4.7%), due to the formation of anoxic zones within the membrane biofilm. Members of the phylum *Firmicutes* were previously reported to be dominant biofilm communities on membrane surfaces of anaerobic reactors (Calderón et al., 2011). With the formation of different redox zones, new niches were created as the membrane biofilm aged. A similar finding was reported for a membrane biofilm in a nitrifying MBR, where autotrophic nitrifiers, dominant in MLSS, were selectively attached to the membrane at the early stage, and later were replaced by the fast-growing and metabolically diverse heterotrophs as the biofilm aged (Lu et al., 2016). As a consequence, key initial colonizer taxa could be inherently predicted, whereas the prediction of aged membrane biofilm community composition was more challenging as the biofilm community significantly diverged from the MLSS over time (Luo et al., 2017). At lower flux, membrane biofilm formation seemed to follow a natural process of colonization and biofilm development (Huang et al., 2008).

Beta diversity revealed a clear difference in the bacterial community between early and relatively aged membrane biofilms (Figure 2 and Supplementary Table S2). This difference was due to the difference in the ecological assembly mechanisms of different-aged membrane biofilms as revealed by the phylogenetic bin-based null model (iCAMP; Ning et al., 2020). Homogenous selection was the most dominant ecological principle – where selection refers to ecological fitness and ongoing abiotic and biotic interaction shaping the community in early (days 1 and 10) membrane biofilms (Figure 4). Previous studies also observed that species sorting by local environmental conditions deterministically shape biofilm communities, rather than being shaped by stochastic dispersal from the suspended biomass, in natural ecosystem like streams (Besemer et al., 2012; Wilhelm et al., 2013), and engineered ecosystems such as MBRs (Matar et al., 2017; Xu et al., 2019), aerobic granular sludge (Ali et al., 2019), anaerobic digester (Mei et al., 2016), and activated sludge systems (Saunders et al., 2016). In full-scale MBRs, stochastic mechanisms did not drive the biofilm community assembly on virgin membrane surfaces after a short period (5h) of filtration (Matar et al., 2017). As the biofilm aged (days 20 and 30), the relative importance of selection decreased, and it was displaced by stochastic mechanisms (dispersal limitation and drift; Figure 4). The increase in dispersal limitation suggests several possibilities due to the lack of connectivity between different redox conditions in biofilms layers (i.e., oxic, anoxic, and anaerobic), resulting in high compositional turnover (Zhou and Ning, 2017). A recent study reported differences in microbial communities between thin (early) and thick (aged) biofilms in a nitrifying reactor which was primarily caused by deterministic factors (Suarez et al., 2019). In a full-scale aerobic granular sludge plant, the influence of the dispersal (stochastic) process in shaping the bacterial community was higher for a flocculant sludge community compared to granular sludge community in the same bioreactor (Ali et al., 2019). Generally, autotrophic nitrogen removal systems have low diversity compared to engineered systems dominated by heterotrophs (Loreau et al., 2001; Fuhrman, 2009; Saikaly and Oerther, 2011). In another study, the influence of stochasticity increased with granule size (age) in a granular biofilm reactor, and community assembly was mainly influenced by drift, which mostly affected low-abundant community members (Liébana et al., 2019). Some low-abundance species are of high importance to maintaining the structure and function of the membrane biofilm community (Xu et al., 2019). Low-abundance species could increase the relative importance of drift processes in shaping membrane biofilm community assembly as membrane biofilm ages. Fouling control should be focused on eliminating low-abundance fouling bacteria present in the membrane biofilm as also suggested elsewhere (Xu et al., 2019).

In the current study, we measured the relative importance of ecological processes of membrane biofilm communities in a lab-scale MBR operated under a well-controlled environment. Our study suggests that the prediction of aged membrane biofilm community composition is more challenging and variable than expected as it is mainly influenced by stochastics processes. Thus, it could be challenging to devise effective fouling mitigation strategies for aged membrane biofilms. Future studies should be expanded to full-scale MBR systems to get a better insight on community assembly processes under a relevant environment.

## Materials and Methods

### Operation of the Lab-Scale MBR

A lab-scale MBR with a working volume of 20L (Matar et al., 2016) was operated using intermittent cycles of a 30min aerobic phase followed by a 30min anoxic phase (Supplementary Figure S9A), to achieve simultaneous carbon and nitrogen removal (Wang et al., 2013). The operating conditions are summarized in Supplementary Table S3.

The lab-scale MBR was inoculated with activated sludge collected from a local wastewater treatment plant (Al Ruwais district, Jeddah, K.S.A), and was acclimated to synthetic wastewater for 45days under continuous filtration mode using commercial ultrafiltration (UF) HFMs (PALL Corporation). A synthetic wastewater composition was used as described elsewhere (Matar et al., 2016). During the acclimation period, the MBR was operated at a constant flux (10 LMH) with cycles of 9min filtration followed by 1min relaxation (no filtration). COD and ammonium ( ${\mathrm{NH}}_{4}^{+}$ ) concentrations were measured in the influent, while COD, ${\mathrm{NH}}_{4}^{+}$ , nitrate ( ${\mathrm{NO}}_{3}^{-}$ ), and nitrite ( ${\mathrm{NO}}_{2}^{-}$ ) concentrations were measured in the effluent. All measurements were performed using Hach kits (Hach Chemical, United States).

After 45days of acclimation, five different types of UF HFMs were tested in parallel in the same MBR tank; four membrane types were manufactured in our lab (Matar et al., 2016) and the fifth membrane was commercial (PALL Corporation). The characteristics (nominal pore size, contact angle, and surface charge) of the different membranes are provided in Supplementary Table S4. The five different membranes were assembled into identical modules following the same procedure, and 12 hollow-fibers achieved a total membrane surface area of 56.5cm^2^ per module. Four membrane cassettes were designed to fit the five different hollow-fiber modules (Supplementary Figure S9B) that can be submerged into the MBR tank to simultaneously expose different membranes types to the same wastewater and suspended biomass.

Two membrane cassettes, each containing five different membrane modules, were submerged and operated under filtration mode. Each membrane cassette was connected to a multi-channel permeate pump (peristaltic), with each membrane module connected to a channel separately. The membrane modules were operated using an imposed permeate flux of 10 LMH (Supplementary Table S3). In parallel, two identical membrane cassettes, also containing five different types of membrane modules, were inserted in the same MBR tank and operated without permeate flux production (static mode, 0 LMH). Membrane modules were run continuously and triplicate membrane fibers were collected on days 1 and 10 from the same membrane cassette. The sampled membrane fibers were cut from the membrane module for microbial community analysis after each run, and the remaining fiber tips were sealed using epoxy glue to ensure the normal operation of the membrane module. After 20days, membrane fibers were collected again from the same membrane cassette where samples were collected on days 1 and 10, and the entire membrane cassette was removed from the operation. On day 30, we stopped the second pump and sampled the membrane fibers from the other membrane cassette. The pressure gauges were connected to the membrane modules that operated continuously for 30days to have a complete TMP profile of 30days. In addition, 20ml of MLSS was collected in parallel to each membrane-sampling event (days 1, 10, 20, and 30). The sampling time of days 1, 10, 20, and 30 was designed to allow us to study early and relatively aged membrane biofilm.

The TMP was measured uninterruptedly with a pressure transducer (68075–32, Cole-Parmer Instrument Company) and recorded using a data acquisition system connected to a computer (LabVIEW, National Instruments).

### DNA and RNA Extraction and PCR Amplification

DNA and RNA were co-extracted from the membrane biofilm and suspended biomass (MLSS) using the Mobio PowerBiofilm RNA Isolation kit (MO BIO Laboratories, Inc., Carlsbad, CA) with minor modifications. The ALLPrep DNA/RNA Mini Kit (Qiagen, Valencia, CA, United States) was used to separate the extracted DNA from RNA as described elsewhere (Ishii et al., 2013). For the membrane modules, DNA and RNA were extracted in duplicate from three different fibers that were selected randomly from each type of membrane module (POX, PTA, SPTA, SPSU, COM) depending on the operation mode (0 and 10 LMH), and was cut into small pieces of 1cm length. The same DNA extraction procedure was carried out for the MLSS in duplicate. All duplicate genomic DNA samples were pooled together and resulted in 40 membrane samples and 4 MLSS samples that were analyzed. The synthesis of cDNA from RNA included the purification and generation of blunt end cDNA. The quality (A260/A280) and quantity (A260) of the extracted DNA and cDNA were determined with a Nanodrop^®^ 1,000 spectrophotometer (Thermo Fisher Scientific, Waltham, MA).

For each sample (membrane biofilm and MLSS), triplicate PCR reactions were performed in a 25μl reaction volume using the HotStarTaq Plus Master Mix (QIAGEN, Valencia, CA), 0.25μM of each primer, and 20ng of template. Bacterial 16S rRNA genes were amplified using the bacteria-specific forward primer 341F (5'-Adaptor A-Barcode-CA Linker- CCTACGGGNGGCWGCAG-3') and reverse primer 805R (5'-Adaptor B-TC Linker- GACTACHVGGGTATCTAATCC-3'; Klindworth et al., 2013), which targeted the V4 region of the bacterial 16S rRNA gene. PCR was performed using life technologies veritus thermocycler with the following PCR conditions: 94°C for 3min, followed by 28cycles of 94°C for 30s; 53°C for 40s and 72°C for 1min; after which a final elongation step at 72°C for 5min was performed.

### Amplicon Sequencing and Data Processing

All amplicon products from different samples were mixed in equal concentrations, purified using Agencourt Ampure beads (Agencourt Bioscience Corporation, MA, United States), and sequenced on the Roche 454 FLX Titanium genome sequencer (Roche, Indianapolis, IN) according to the manufacturer’s instructions.

The 16S rRNA gene and 16S rRNA amplicons were processed using QIIME (Quantitative Insights Into Microbial Ecology v1.7.0; Caporaso et al., 2010). Initially, all sequences were denoised and filtered for quality checks, and then sequence reads were demultiplexed and barcoded primers were removed. Low-quality reads were removed, including reads below 200 base pairs (bp) and above 1,000bp, reads that contained more than 6 ambiguous bp, and sequences with a quality score below 25. The quality-filtered reads were dereplicated and formatted for use in the UPARSE workflow (Edgar, 2013). The cluster_otus command of usearch (v. 10.0.240) performed 97% OTU clustering using the UPARSE-OTU algorithm. Chimeras were also filtered by the cluster_otus command. Taxonomy was assigned using sintax as implemented in usearch (v. 10.0.240), using the MiDAS database (v.3.6), an ecosystem-specific reference database for activated sludge (Nierychlo et al., 2020). The OTU count and corresponding taxonomy table were imported into the Rstudio IDE environment using the ampvis2 package by the amp_import_usearch command (Albertsen et al., 2015). To remove inherent heterogeneity of sampling depth, we subsampled the dataset (normalized abundance values) to an even depth of 4,386 sequences across the samples. Alpha diversity indices and NMDS of Bray Curtis and Unweighted UniFrac distance matrix were performed using the ampvis2 package. Beta diversity was measured to assess the dissimilarities between two different samples. Bray Curtis distance metrics were used for permutational multivariate analysis of variance (MANOVA or ADONIS) with 999 permutations to test for significant differences in community composition between different categories using vegan package 2.4.3 (Oksanen et al., 2017).

### iCAMP Analysis to Quantify the Community Assembly Mechanism

The importance of various ecological processes in community assembly was quantitatively assessed using the iCAMP package in the Rstudio IDE environment as described elsewhere (Ning et al., 2020). The iCAMP approach is more accurate, precise, and sensitive than other approaches such as NP (Ning et al., 2019) and QPEN (Stegen et al., 2013, 2015). iCAMP provides a robust tool to quantify microbial assembly processes. This is based on a quantitative framework that describes assembly processes in terms of selection (heterogeneous or homogenous), dispersal [dispersal limitation, or homogenizing dispersal (mass effects)], and/or drift. Briefly, to quantify various ecological processes, the observed taxa are first divided into different groups (called “bins”) based on their phylogenetic relationships. Then, the process governing each bin is identified based on null model analysis of the phylogenetic diversity using β Net Relatedness Index (βNRI) and taxonomic β-diversities using the modified Raup-Crick metric (RC). For each bin, the fraction of pairwise comparisons with βNRI was performed and considered as assembled by heterogenous $\left(\beta NRI>+1.96\right)$ or homogenous $\left(\beta NRI>-1.96\right)$ selection processes. If the difference was not significant, the observed differences in phylogenetic composition were considered to be the result of dispersal or drift. These were differentiated using the RC dissimilarity metric. If RC>+0.95 , assembly was considered to be the result of dispersal limitation coupled with drift; if RC<−0.95, then bin assembly was considered to be the result of homogenizing dispersal mechanisms; and if RC was between −0.95 and +0.95, then the observed differences in phylogenetic composition were considered to be the result of random processes (drift). The above analysis is repeated for every bin. Subsequently, the fractions of individual processes across all bins are weighted by each bins relative abundance and summarized to estimate the relative importance of individual processes at the whole community level.

## Data Availability Statement

The datasets presented in this study can be found in online repositories. The names of the repository/repositories and accession number(s) can be found at: https://www.ncbi.nlm.nih.gov/bioproject/PRJNA631865.

## Author Contributions

GM, MA, SB, SN, W-TL, and PS planned the research. GM designed and executed the experiments and MA analysed the data and performed iCAMP analysis. GM, MA, and PS wrote the paper with critical feedback from all authors. All authors contributed to the article and approved the submitted version.

## Funding

This work was supported by Academic Partnership Program (APP) and Center Competitive Funding Program (FCC/1/1971-05-01) from King Abdullah University of Science and Technology (KAUST).

## Conflict of Interest

The authors declare that the research was conducted in the absence of any commercial or financial relationships that could be construed as a potential conflict of interest.

## Publisher’s Note

All claims expressed in this article are solely those of the authors and do not necessarily represent those of their affiliated organizations, or those of the publisher, the editors and the reviewers. Any product that may be evaluated in this article, or claim that may be made by its manufacturer, is not guaranteed or endorsed by the publisher.
