# Supplementary material for: Relative Importance of Stochastic Assembly Process of Membrane Biofilm Increased as Biofilm Aged
Source: Front Microbiol. 2021 Sep 10;12:708531. doi: 10.3389/fmicb.2021.708531 (PMC8461090; doi:10.3389/fmicb.2021.708531)
Supplement: Supplementary file 1 [file Data_Sheet_1.PDF]

Supplementary Material

Relative importance of stochastic assembly process of membrane biofilm increased as biofilm aged

Gerald K. Matar[a,1], Muhammad Ali[a,1], Samik Bagchi[a,2], Suzana Nunes[b], Wen-Tso Liu[c], Pascal E. Saikaly[a] *

[a]King Abdullah University of Science and Technology, Biological and Environmental Science and Engineering Division, Water Desalination and Reuse Research Center, Thuwal 23955-6900, Saudi Arabia

[b]King Abdullah University of Science and Technology, Biological and Environmental Science and Engineering Division, Advanced Membranes and Porous Materials Center, Thuwal 23955-6900, Saudi Arabia

[c]University of Illinois at Urbana-Champaign, Department of Civil and Environmental Engineering, 3207 Newmark Civil Engineering Laboratory, 205 N. Mathews Ave., Urbana, IL 61801, USA

*Corresponding author: Pascal E. Saikaly, pascal.saikaly@kaust.edu.sa

[1]G.K.M and M.A. contributed equally to this work.

[2]Present address: Digested Organics, 23745 Research Drive, Farmington Hills, MI 48335, USA

1 Supplementary Figures and Tables

1.1 Supplementary Figures

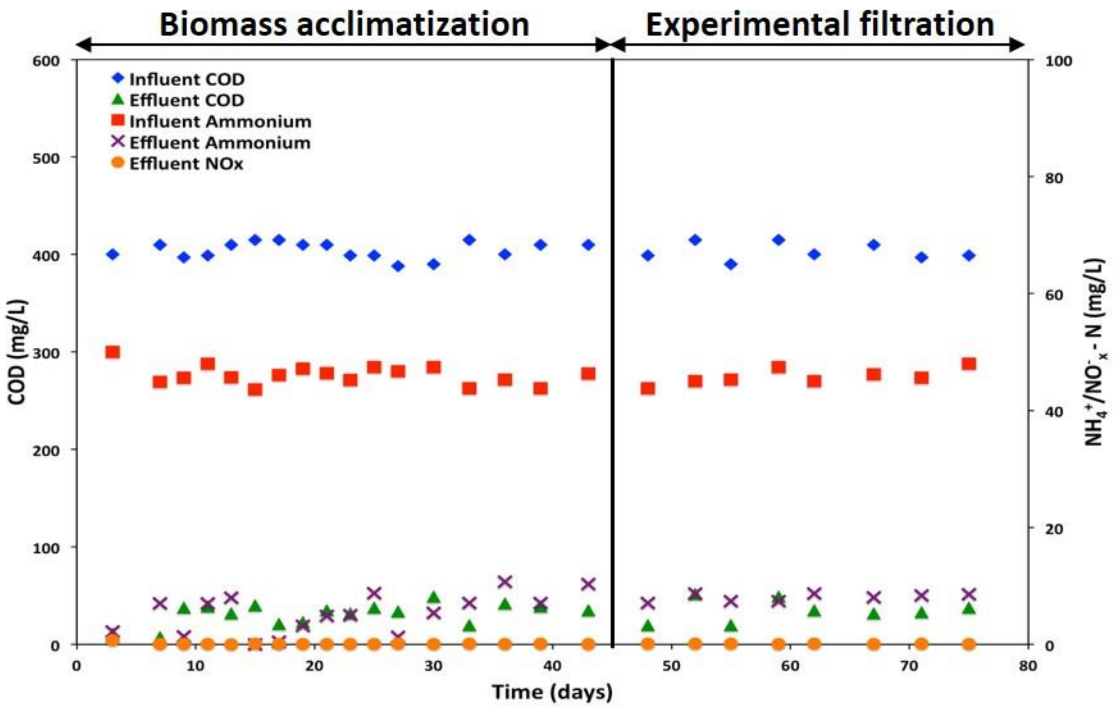

Fig. S1. Fig. S1. COD and NH_4^+ concentrations in the synthetic wastewater influent and COD, NH_4^+, NO_2^-, and NO_3^- concentrations in the wastewater effluent.

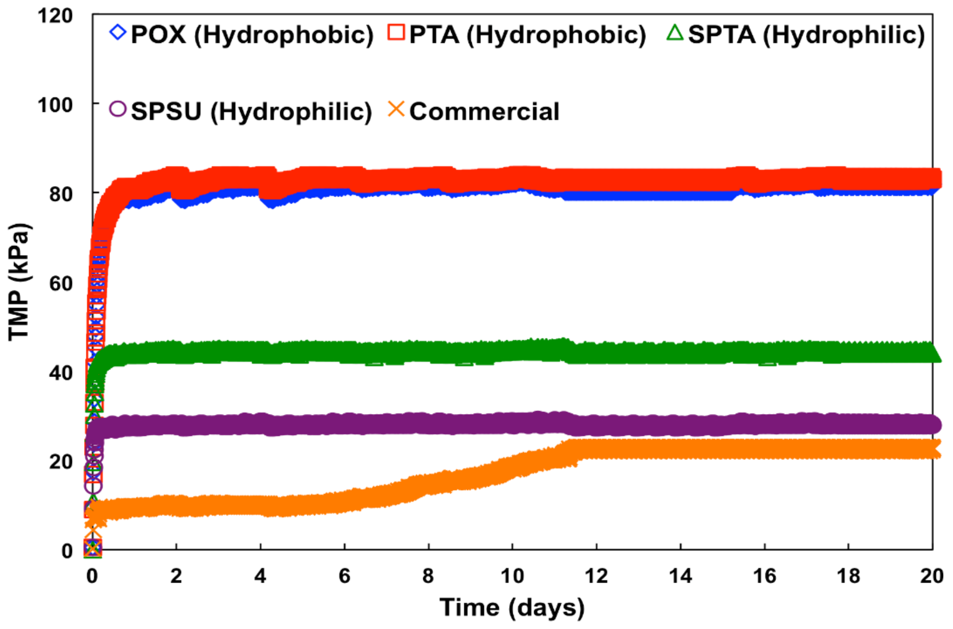

Fig. S2. Transmembrane pressure (TMP) profiles for the five membranes during filtration. The TMPs remained at this level till the end of the experiment.

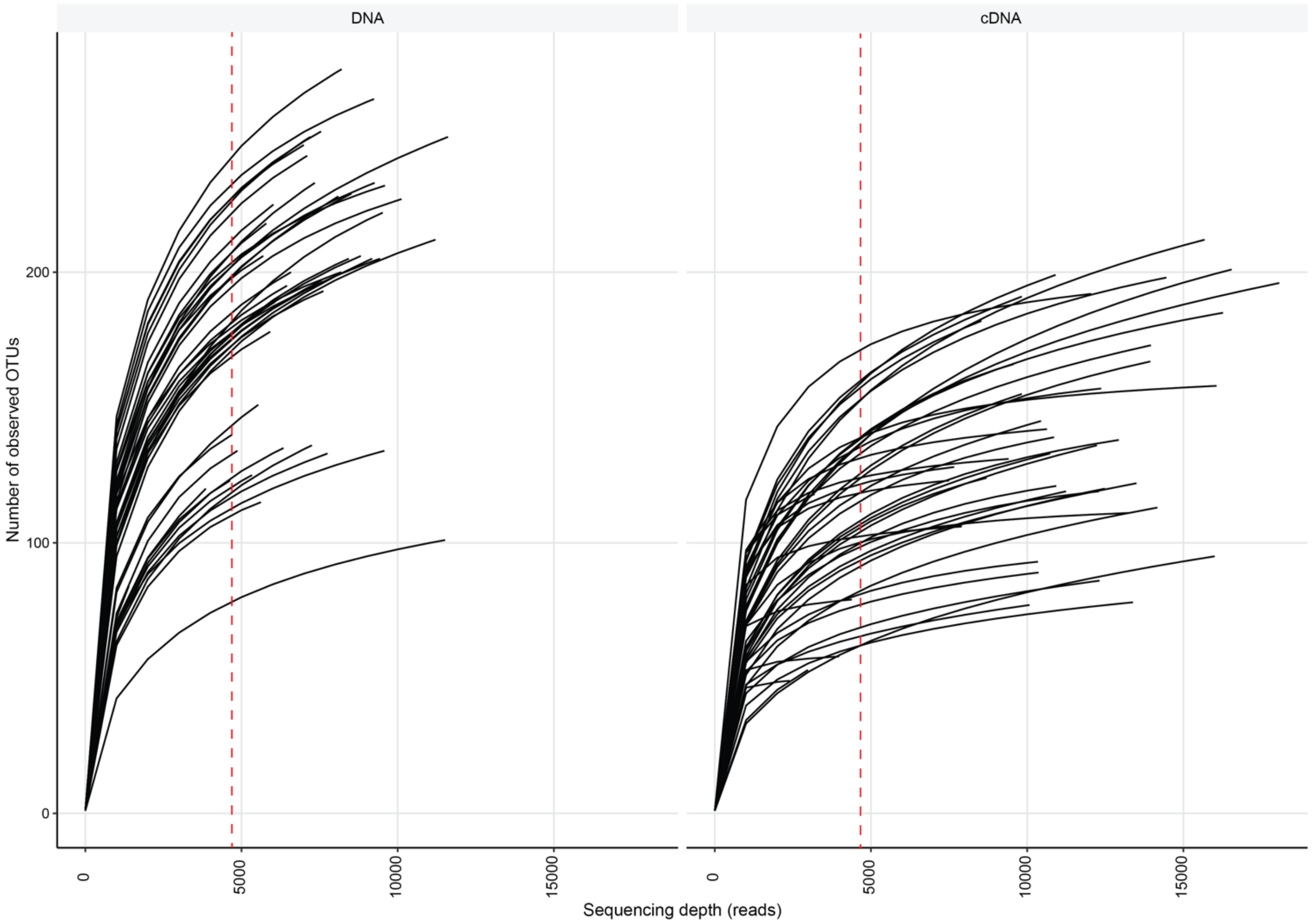

Fig. S3: Sample-based rarefaction curves (number of reads vs number of observed OTUs). The samples were rarefied to an even number of reads (4,386) per sample represented as red dashed lines.

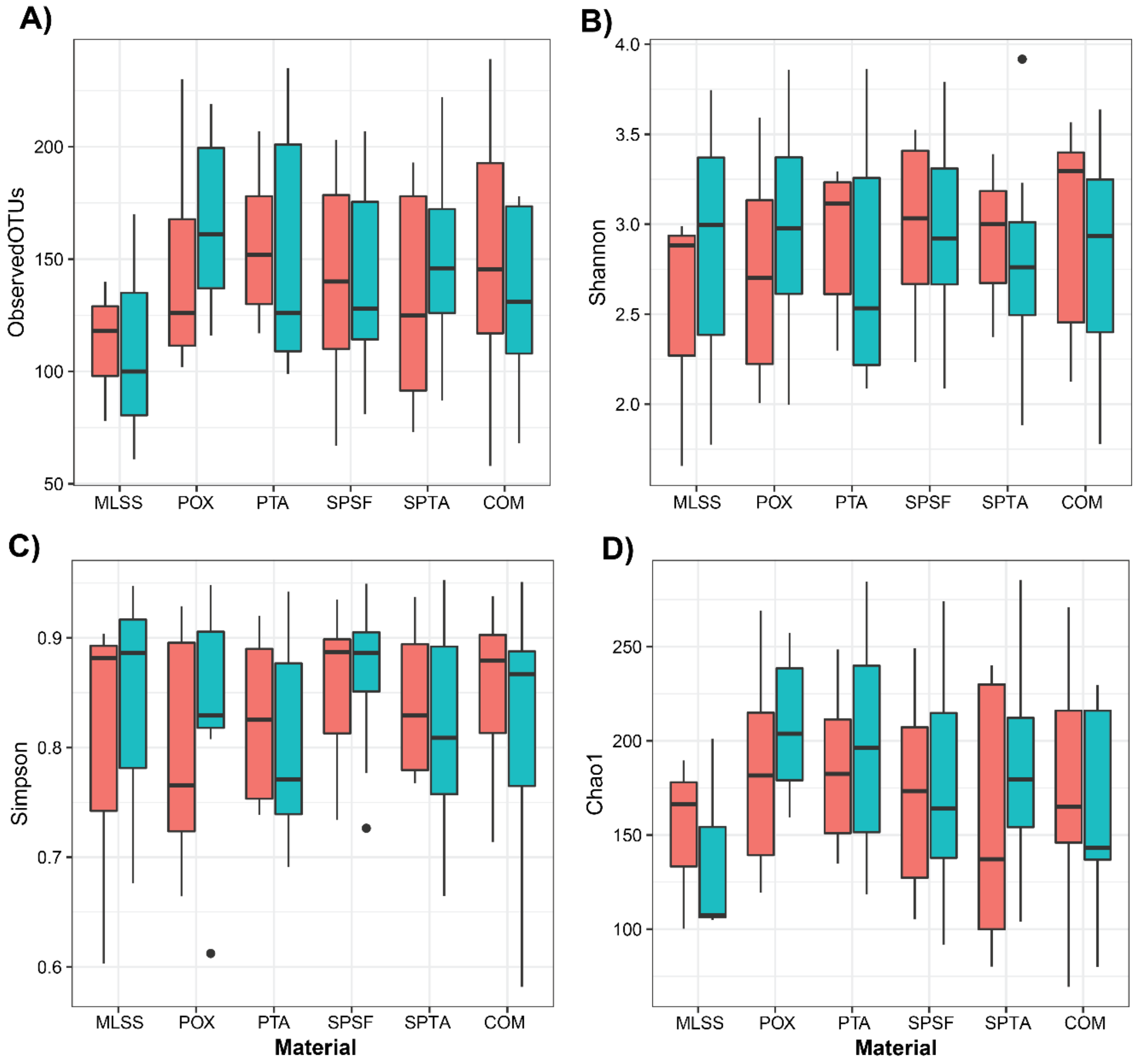

Fig. S4. Microbial community diversity according to variances in 16S rRNA gene from both DNA and cDNA measured using alpha diversity measures. Four different richness estimators were calculated based on a rarefied sub-sampled to 4,386 sequences per sample. Red and green boxplots represent dataset from DNA and cDNA samples, respectively. The upper and lower bounds of boxes denote the 25th and 75th percentiles and the lines indicate the max and min values, respectively. The dataset used in this analysis includes both flux and no flux conditions. Outliers are shown as dots.

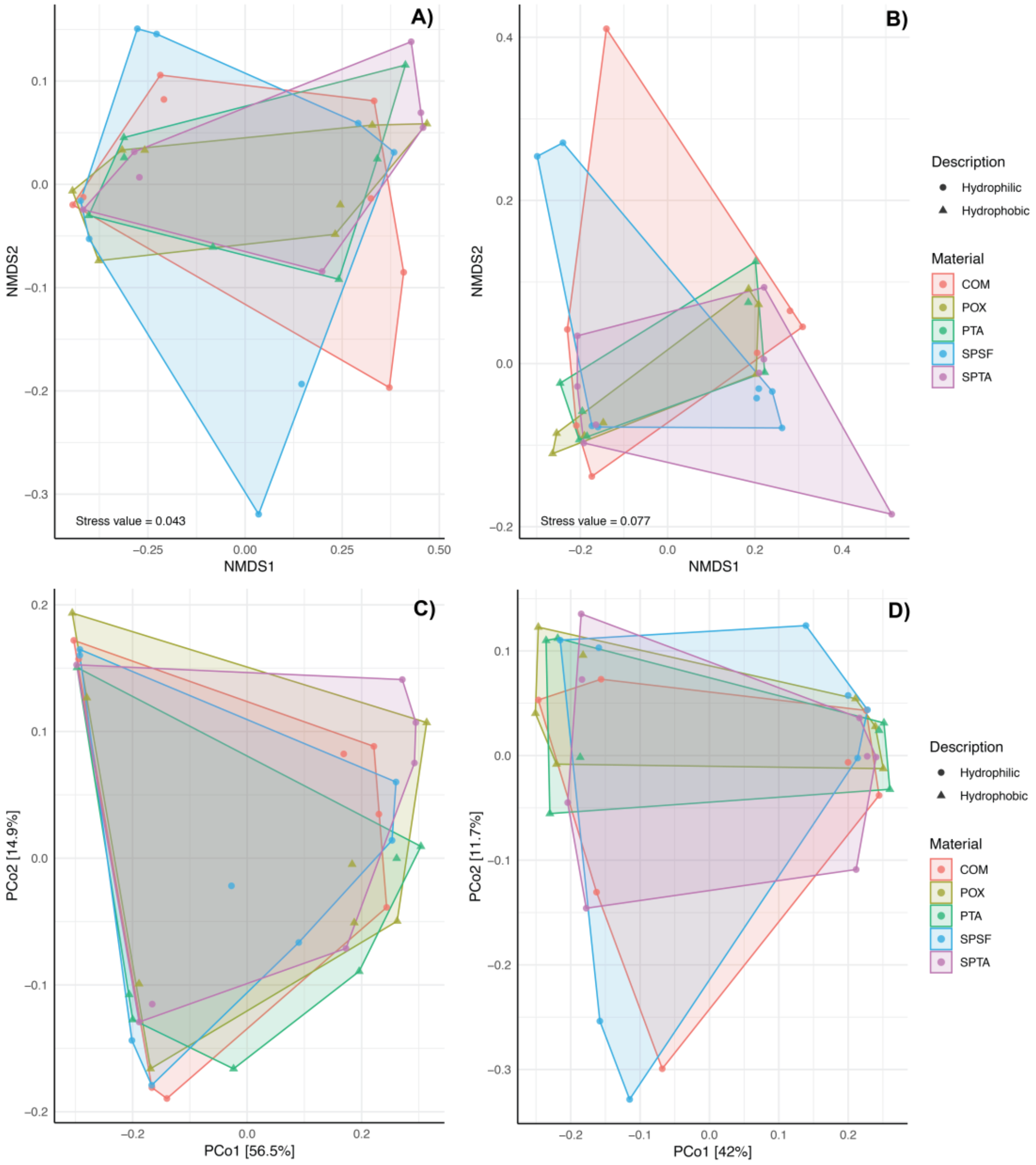

Fig. S5. The ordination plots of membrane biofilms formed on different membrane materials such as POX (polyoxadiazole), PTA (polytriazole), SPTA (sulfonated polytriazole), SPSU (sulfonated polysulfone) and COM (commercial PVDF (poly(vinylidene fluoride)) membranes. Non-metric Multidimensional Scaling (NMDS) analysis based on Bray-Curtis **(A & B)** and Principal Coordinates Analysis (PCoA) analysis based on Unweighted UniFrac **(C & D)** distance metrics. Panels **A & C** represent 16S rDNA and **B & D** represent 16S rRNA dataset. These plots were generated based on

rarefied dataset sub-sampled to 4,386 sequences per sample. The dataset used in this analysis includes both flux and no flux conditions.

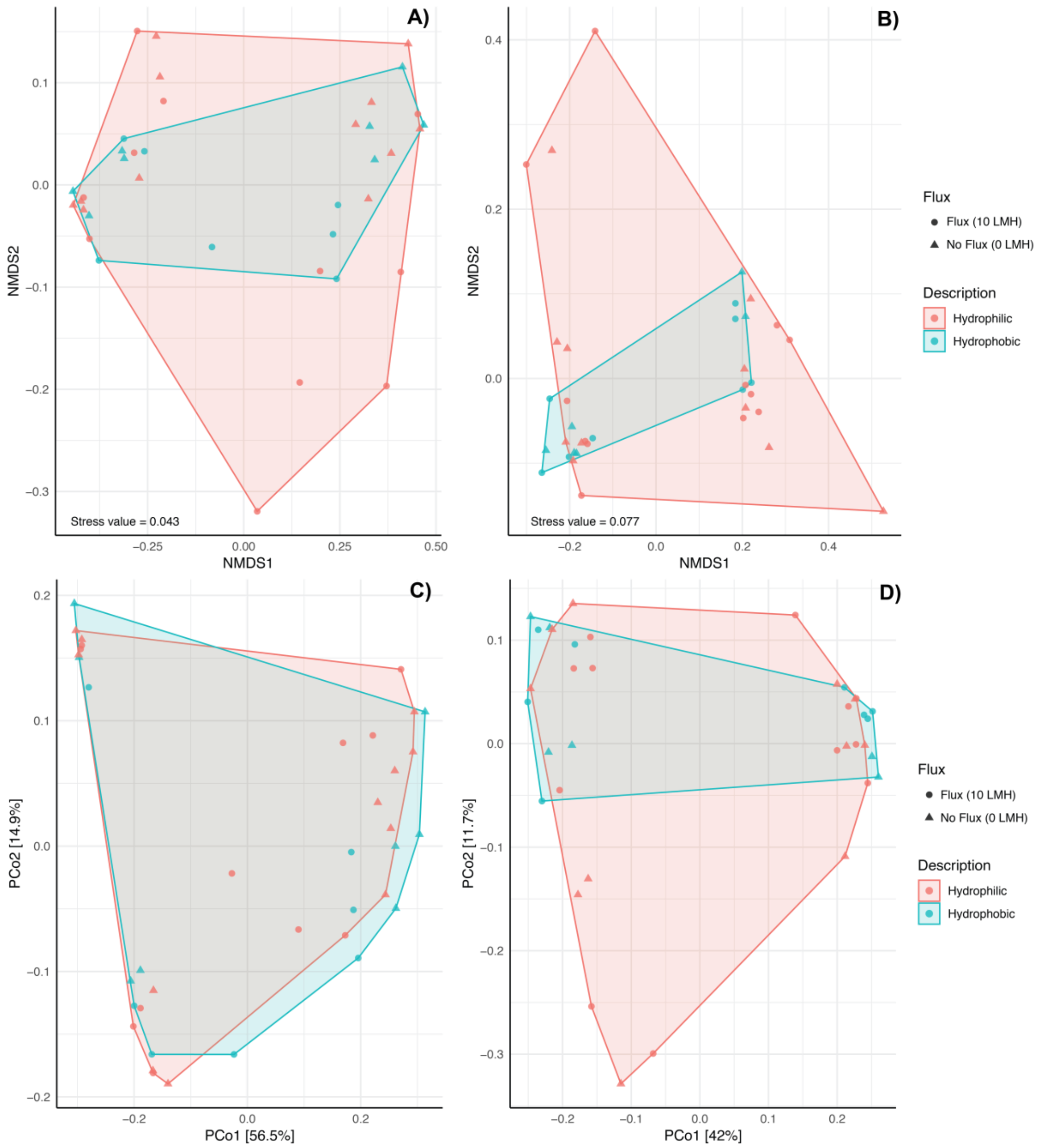

Fig. S6. The ordination plots of biofilms harvested from hydrophilic and hydrophobic membrane surfaces. Non-metric Multidimensional Scaling (NMDS) analysis based on Bray-Curtis **(A & B)** and Principal Coordinates Analysis (PCoA) analysis based on Unweighted UniFrac **(C & D)** distance metrics. Panels **A & C** represent 16S rDNA and **B & D** represent 16S rRNA dataset. These plots were generated based on rarefied dataset sub-sampled to 4,386 sequences per sample.

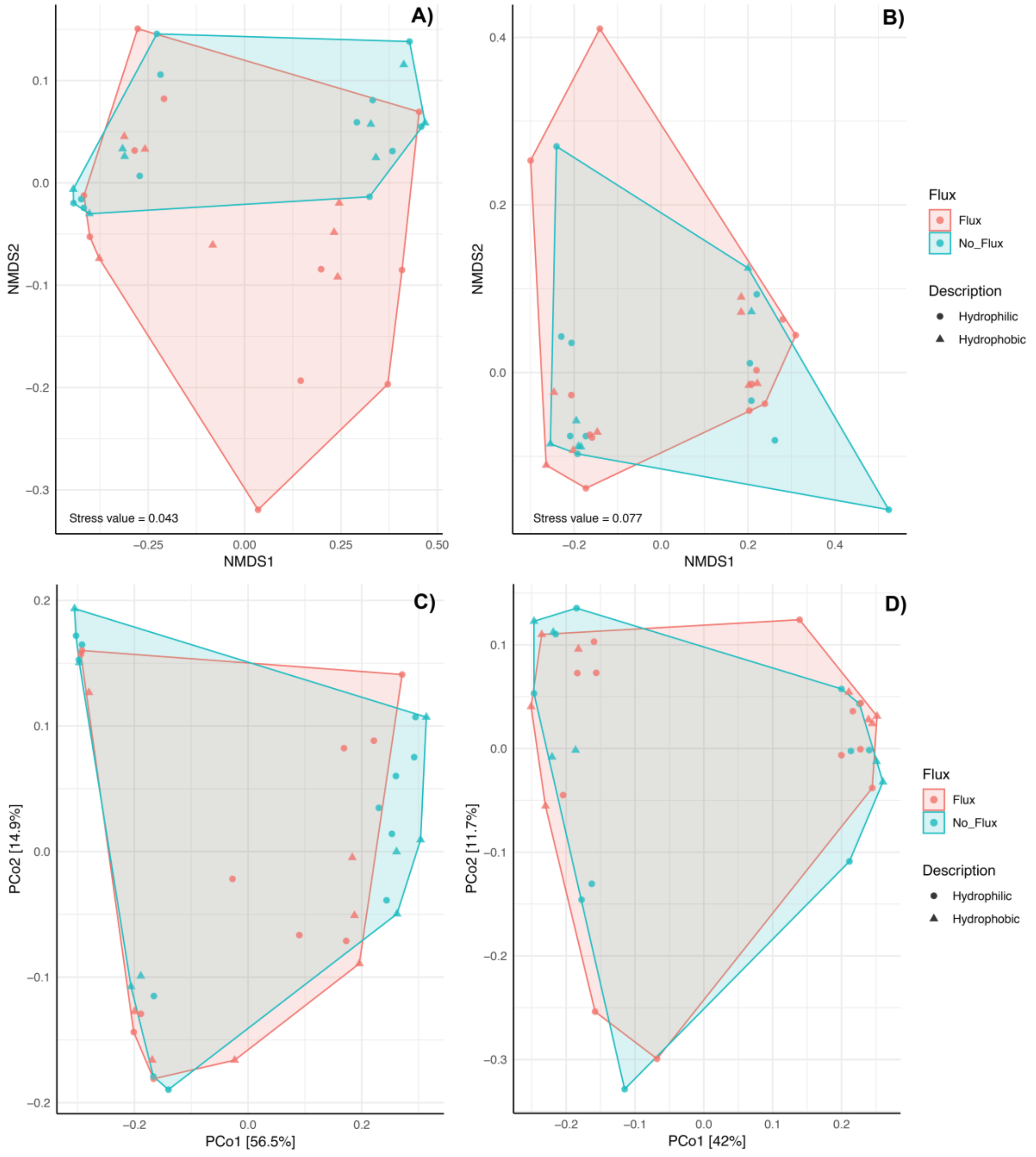

Fig. S7. The ordination plots of membrane biofilms operated with a permeate flux of 10 LMH and without permeate production (0 LMH). Non-metric Multidimensional Scaling (NMDS) analysis based on Bray-Curtis **(A & B)** and Principal Coordinates Analysis (PCoA) analysis based on Unweighted UniFrac **(C & D)** distance metrics. Panels **A & C** represent 16S rDNA and **B & D** represent 16S rRNA dataset. These plots were generated with rarefied dataset sub-sampled to 4,386 sequences per sample.

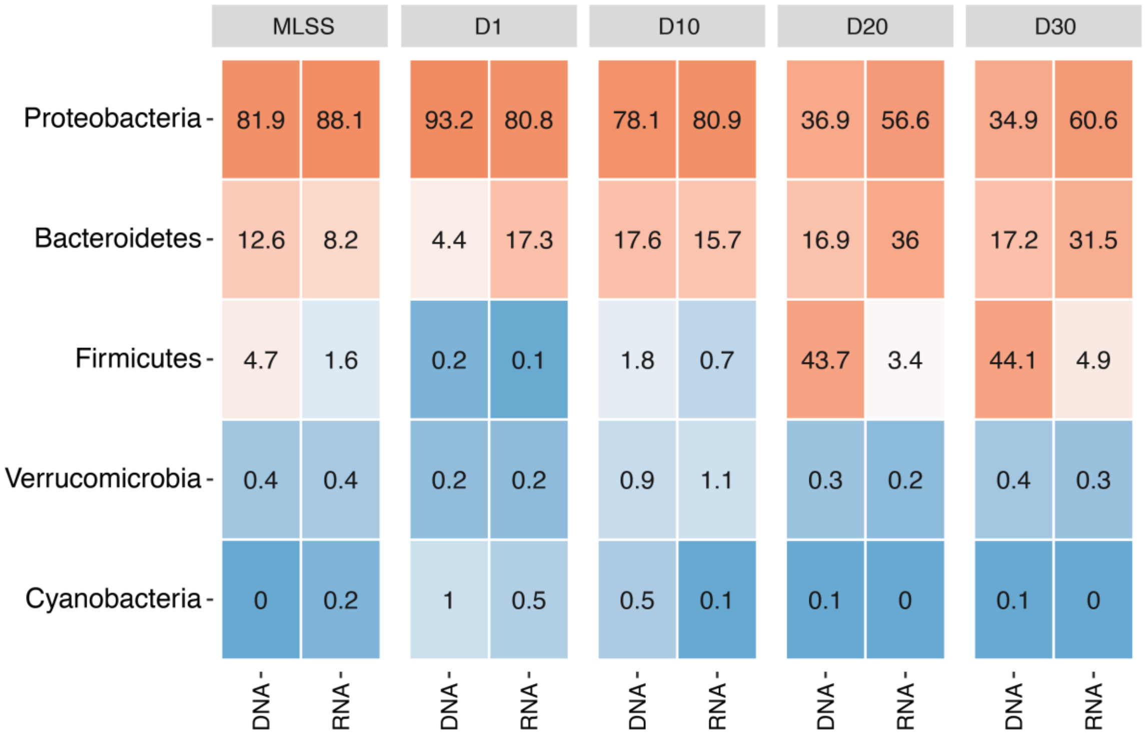

Fig. S8. Heatmap distribution of phylum-level classification derived average relative reads abundance for mixed liquor suspended sludge and membrane biofilms. MLSS corresponds to the mixed liquor suspended solids sample. D1, D10, D20, and D30 refer to sampling days 1, 10, 20, and 30, respectively. The color intensity in each cell reflects relative reads abundance of taxa in the corresponding sample. Similarly, DNA and RNA represent 16S rRNA amplicon sequencing dataset from DNA and cDNA, respectively. The heatmap generated based on a rarefied dataset sub-sampled to 4,386 sequences per sample. The dataset used in this analysis includes both flux and no flux conditions.

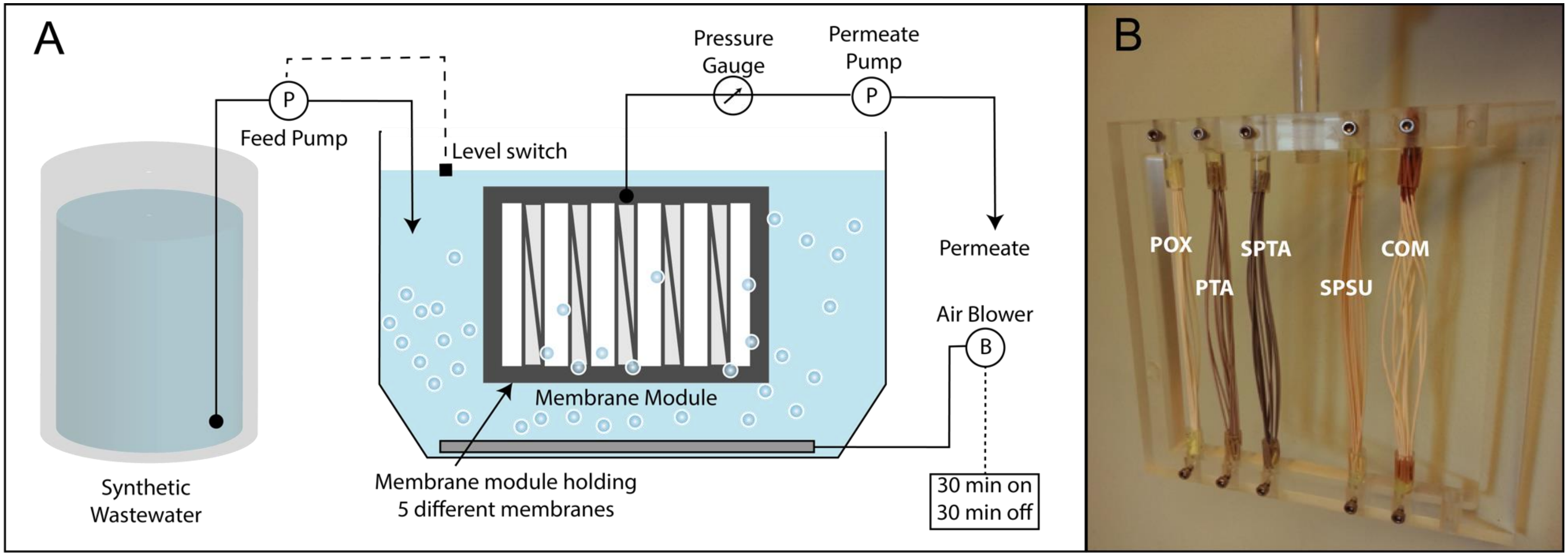

Fig. S9. A) Schematic diagram of the lab-scale membrane bioreactor. **B)** Membrane cassette designed to simultaneously hold five different hollow-fiber membrane modules.

1.2 Supplementary Tables

Table S1. Metadata of the sequenced samples.

S. No.	Sample ID	Description	Material[1]	Sample Type	Flux[2]	Time (d)	No. of Reads
1	MLSSD10DNA	MLSS	MLSS	DNA	MLSS	10	2
2	SPTAFD1DNA	Hydrophilic	SPTA	DNA	F	1	1392
3	POXNFD30RNA	Hydrophobic	POX	RNA	NF	30	2395
4	MLSSD10RNA	MLSS	MLSS	RNA	MLSS	10	2981
5	PTANFD30RNA	Hydrophobic	PTA	RNA	NF	30	3193
6	PTAFD1DNA	Hydrophobic	PTA	DNA	F	1	3856
7	COMNFD30RNA	Hydrophilic	COM	RNA	NF	30	3980
8	SPTANFD30RNA	Hydrophilic	SPTA	RNA	NF	30	4386
9	COMFD1DNA	Hydrophilic	COM	DNA	F	1	4633
10	POXFD1DNA	Hydrophobic	POX	DNA	F	1	4703
11	SPTANFD1DNA	Hydrophilic	SPTA	DNA	NF	1	4868
12	PTANFD1DNA	Hydrophobic	PTA	DNA	NF	1	5336
13	MLSSD1DNA	MLSS	MLSS	DNA	MLSS	1	5536
14	POXNFD1DNA	Hydrophobic	POX	DNA	NF	1	5619
15	COMNFD10DNA	Hydrophilic	COM	DNA	NF	10	5695
16	SPSFNFD30DNA	Hydrophilic	SPSF	DNA	NF	30	5791
17	POXNFD10DNA	Hydrophobic	POX	DNA	NF	10	5918
18	PTANFD30DNA	Hydrophobic	PTA	DNA	NF	30	6020

S. No.	Sample ID	Description	Material[1]	Sample Type	Flux[2]	Time (d)	No. of Reads
19	SPSFFD20DNA	Hydrophilic	SPSF	DNA	F	20	6069
20	SPSFFD1DNA	Hydrophilic	SPSF	DNA	F	1	6343
21	SPSFNFD10DNA	Hydrophilic	SPSF	DNA	NF	10	6455
22	SPTANFD10DNA	Hydrophilic	SPTA	DNA	NF	10	6583
23	SPSFFD10DNA	Hydrophilic	SPSF	DNA	F	10	6990
24	POXNFD30DNA	Hydrophobic	POX	DNA	NF	30	6992
25	POXFD30DNA	Hydrophobic	POX	DNA	F	30	7102
26	SPTAFD30DNA	Hydrophilic	SPTA	DNA	F	30	7205
27	COMNFD1DNA	Hydrophilic	COM	DNA	NF	1	7252
28	POXFD20DNA	Hydrophobic	POX	DNA	F	20	7346
29	POXNFD20RNA	Hydrophobic	POX	RNA	NF	20	7499
30	PTAFD30DNA	Hydrophobic	PTA	DNA	F	30	7545
31	SPTAFD10DNA	Hydrophilic	SPTA	DNA	F	10	7569
32	PTANFD10DNA	Hydrophobic	PTA	DNA	NF	10	7620
33	PTANFD20RNA	Hydrophobic	PTA	RNA	NF	20	7662
34	SPSFNFD1DNA	Hydrophilic	SPSF	DNA	NF	1	7752
35	SPTANFD20RNA	Hydrophilic	SPTA	RNA	NF	20	7904
36	SPTANFD30DNA	Hydrophilic	SPTA	DNA	NF	30	8099
37	COMFD10DNA	Hydrophilic	COM	DNA	F	10	8196
38	PTAFD20DNA	Hydrophobic	PTA	DNA	F	20	8204

S. No.	Sample ID	Description	Material[1]	Sample Type	Flux[2]	Time (d)	No. of Reads
39	COMFD20DNA	Hydrophilic	COM	DNA	F	20	8434
40	COMNFD20DNA	Hydrophilic	COM	DNA	NF	20	8516
41	COMNFD20RNA	Hydrophilic	COM	RNA	NF	20	8535
42	POXNFD10RNA	Hydrophobic	POX	RNA	NF	10	8699
43	SPTANFD20DNA	Hydrophilic	SPTA	DNA	NF	20	8818
44	PTAFD10DNA	Hydrophobic	PTA	DNA	F	10	9180
45	COMNFD30DNA	Hydrophilic	COM	DNA	NF	30	9241
46	PTANFD20DNA	Hydrophobic	PTA	DNA	NF	20	9259
47	SPSFNFD30RNA	Hydrophilic	SPSF	RNA	NF	30	9406
48	POXNFD20DNA	Hydrophobic	POX	DNA	NF	20	9432
49	COMFD30DNA	Hydrophilic	COM	DNA	F	30	9520
50	SPTAFD10RNA	Hydrophilic	SPTA	RNA	F	10	9562
51	MLSSD30DNA	MLSS	MLSS	DNA	MLSS	30	9573
52	SPSFFD30DNA	Hydrophilic	SPSF	DNA	F	30	9590
53	SPSFFD20RNA	Hydrophilic	SPSF	RNA	F	20	9827
54	SPTAFD30RNA	Hydrophilic	SPTA	RNA	F	30	9827
55	SPSFNFD10RNA	Hydrophilic	SPSF	RNA	NF	10	10075
56	SPSFNFD20DNA	Hydrophilic	SPSF	DNA	NF	20	10119
57	SPSFFD10RNA	Hydrophilic	SPSF	RNA	F	10	10344
58	SPTANFD10RNA	Hydrophilic	SPTA	RNA	NF	10	10363

S. No.	Sample ID	Description	Material[1]	Sample Type	Flux[2]	Time (d)	No. of Reads
59	POXFD1RNA	Hydrophobic	POX	RNA	F	1	10441
60	COMFD20RNA	Hydrophilic	COM	RNA	F	20	10633
61	PTAFD1RNA	Hydrophobic	PTA	RNA	F	1	10739
62	COMNFD10RNA	Hydrophilic	COM	RNA	NF	10	10859
63	SPSFNFD20RNA	Hydrophilic	SPSF	RNA	NF	20	10907
64	SPSFNFD1RNA	Hydrophilic	SPSF	RNA	NF	1	10929
65	SPTAFD20DNA	Hydrophilic	SPTA	DNA	F	20	11209
66	PTAFD10RNA	Hydrophobic	PTA	RNA	F	10	11240
67	MLSSD20DNA	MLSS	MLSS	DNA	MLSS	20	11515
68	POXFD10DNA	Hydrophobic	POX	DNA	F	10	11609
69	MLSSD1RNA	MLSS	MLSS	RNA	MLSS	1	12047
70	PTANFD1RNA	Hydrophobic	PTA	RNA	NF	1	12231
71	COMFD1RNA	Hydrophilic	COM	RNA	F	1	12295
72	COMFD10RNA	Hydrophilic	COM	RNA	F	10	12316
73	PTANFD10RNA	Hydrophobic	PTA	RNA	NF	10	12368
74	SPSFFD1RNA	Hydrophilic	SPSF	RNA	F	1	12486
75	POXNFD1RNA	Hydrophobic	POX	RNA	NF	1	12932
76	MLSSD30RNA	MLSS	MLSS	RNA	MLSS	30	13237
77	COMNFD1RNA	Hydrophilic	COM	RNA	NF	1	13385
78	SPTAFD1RNA	Hydrophilic	SPTA	RNA	F	1	13498

S. No.	Sample ID	Description	Material[1]	Sample Type	Flux[2]	Time (d)	No. of Reads
79	PTAFD20RNA	Hydrophobic	PTA	RNA	F	20	13941
80	POXFD10RNA	Hydrophobic	POX	RNA	F	10	13960
81	SPTANFD1RNA	Hydrophilic	SPTA	RNA	NF	1	14165
82	POXFD20RNA	Hydrophobic	POX	RNA	F	20	14455
83	PTAFD30RNA	Hydrophobic	PTA	RNA	F	30	15678
84	MLSSD20RNA	MLSS	MLSS	RNA	MLSS	20	16001
85	COMFD30RNA	Hydrophilic	COM	RNA	F	30	16062
86	SPTAFD20RNA	Hydrophilic	SPTA	RNA	F	20	16267
87	POXFD30RNA	Hydrophobic	POX	RNA	F	30	16544
88	SPSFFD30RNA	Hydrophilic	SPSF	RNA	F	30	18070

[1]MLSS (Mixed Liquor Suspended Solids), POX (polyoxadiazole), PTA (polytriazole), SPTA (sulfonated polytriazole), SPSU (sulfonated polysulfone) and COM (commercial PVDF membrane)

[2]F (operated with 10 LMH flux), NF (operated with 0 LMH flux)

Table S2. Permutational MANOVA (ADONIS) analysis based on Bray–Curtis distance between membrane biofilm of different age (1, 10, 20 and 30 days).

Comparison	Bray–Curtis R^2		
	**All Samples**	**16S rDNA only**	**16S rRNA only**
Day 1 VS Day 10	0.20773	0.53732	0.26778
Day 1 VS Day 20	0.50635	0.71041	0.68037
Day 1 VS Day 30	0.55261	0.8034	0.69101
Day 10 VS Day 20	0.39086	0.6387	0.5384
Day 10 VS Day 30	0.41001	0.71869	0.51741
Day 20 VS Day 30	0.03317	0.0573	0.15839

Significance in the ADONIS R^2 statistic is based on 999 randomizations; P-values were < 0.001 for all analyses.

Table S3. Operating conditions of the lab-scale MBR.

	**Aerobic phase**	**Anoxic phase**
DO (mg/L)	8.60 ± 0.19	0.14 ± 0.07
pH	7.80 ± 0.20	7.67 ± 0.19
Conductivity (mS)	3.09 ± 0.48	3.10 ± 0.44
SRT (d)	15	
HRT (h)	12	
Flux (L/m^2/h)	10	
Reactor volume (L)	20	
Aeration cycle	30 min Aerobic / 30 min Anoxic	
Aeration flow rate	2 L/min	
Operation	9 min filtration / 1 min relaxation	
MLSS (mg/L)	3743 ± 311	
MLVSS (mg/L)	3106 ± 291	
MLVSS / MLSS	0.83 ± 0.04	
Duration of operation (days)	Phase I: 45 d, biomass acclimatization<br>Phase II: 30 d, continuous filtration	

Table S4. Membrane properties and surface characteristics.

		Membrane properties			
**Membrane type**	**Acronym**	**Nominal pore size (μm)**	**Contact Angle (º)**	**Zeta potential (mV)**	**Polymer composition and characteristics**
Polyoxadiazole	POX	0.1	96.4±3.2	-28±1	Very hydrophobic membrane (fluorinated)
Polytriazole	PTA	0.1	85.2±12.0	-31±1	Hydrophobic membrane (fluorinated)
Sulfonated Polytriazole	SPTA	0.1	65.4±7.5	-23±1	Hydrophilic membrane (sulfonic group)
Sulfonated Polysulfone	SPSU	0.1	54.5±3.9	-106±1	Hydrophilic membrane (Sulfonic group)
Commercial Membrane	COM	0.1	n.a.*	n.a.*	Commercial membrane (PVDF) (fluorinated)

*n.a. Not available
